# Whole-cell biocatalysts by design

**DOI:** 10.1186/s12934-017-0724-7

**Published:** 2017-06-13

**Authors:** Baixue Lin, Yong Tao

**Affiliations:** CAS Key Laboratory of Microbial Physiological and Metabolic Engineering, Institute of Microbiology, Chinese Academy of Sciences, Beijing, 100101 People’s Republic of China

**Keywords:** Whole-cell biocatalysis, Design, Optimization, Biosynthetic pathway, Cofactor balance, Metabolic engineering, Synthetic biology

## Abstract

Whole-cell biocatalysts provide unique advantages and have been widely used for the efficient biosynthesis of value-added fine and bulk chemicals, as well as pharmaceutically active ingredients. What is more, advances in synthetic biology and metabolic engineering, together with the rapid development of molecular genetic tools, have brought about a renaissance of whole-cell biocatalysis. These rapid advancements mean that whole-cell biocatalysts can increasingly be rationally designed. Genes of heterologous enzymes or synthetic pathways are increasingly being introduced into microbial hosts, and depending on the complexity of the synthetic pathway or the target products, they can enable the production of value-added chemicals from cheap feedstock. Metabolic engineering and synthetic biology efforts aimed at optimizing the existing microbial cell factories concentrate on improving heterologous pathway flux, precursor supply, and cofactor balance, as well as other aspects of cellular metabolism, to enhance the efficiency of biocatalysts. In the present review, we take a critical look at recent developments in whole-cell biocatalysis, with an emphasis on strategies applied to designing and optimizing the organisms that are increasingly modified for efficient production of chemicals.

## Background

Developing sustainable bio-based processes for production of fuels, chemicals, and materials is increasingly attractive due to increased concern for the environment. Chemical synthesis processes are generally high-yield, however they are often environmentally unfriendly and are associated with the production of unwanted byproducts, thus reducing efficiency and increasing downstream costs. In comparison with chemical catalysis, whole-cell biocatalysis offer some unique advantages (Table 1) and provides an efficient and environmentally friendly alternative to traditional chemical synthesis for production of bulk and fine chemicals [1, 2]. In the past several decades, many biocatalytic processes have been implemented to produce a wide variety of products in various industries [3, 4]. The most important advantage of a biocatalyst is its high selectivity. The high selectivity, including regio-, chemo-, diastereo- and enantioselectivity, is very desirable in chemical synthesis and produces benefits such as reduced (or zero) use of protecting groups, minimized side reactions, easier separation of products, and fewer environmental problems [5]. Other advantages such as multi-step reactions in single strain with cofactor regeneration; high catalytic efficiency and mild conditions are also very attractive in commercial applications. In some cases, such as the asymmetric synthesis of chiral target compounds or the synthesis of some sophisticated chemicals, generating the desired products by traditional chemical means is challenging, and biotransformations can be the solution to these challenges. In addition, according to the FDA and European legislation, products obtained by biotechnological methods can also be considered natural, if the substrate for the process is of natural origin [6]. The label of natural is important for the profitability of bioprocess produced products. For example, 2-phenylethanol (2-PE) obtained by chemical synthesis from benzene or styrene is of a price about US$5/kg. However, natural 2-PE is about US$ 1000/kg [7]. By far, 20.4–26.5 g/L 2-PE (space–time yields of 0.3 g/L/h) can be achieved in bioprocess [8, 9]. The yield of bioprocess may be not as high as chemical synthesis (yield >98%) [10]. The label of “natural” and difference in price of a natural compound and its chemically synthesized counterpart can be considerable. Therefore, whole-cell bioprocess holds promise to be the commercially viable route to produce compounds that used in food, beverages, and cosmetics field. The most common drawbacks of biocatalysts include the presence of substrate or product inhibition, the presence of metabolic by-products, and the membrane acting as a mass transport barrier. A biocatalyst can be tailored with protein engineering and metabolic engineering methods to cope with these constraints. Nonetheless, conventional chemical synthesis still remains the staple of the chemical and pharmaceutical industries by far. The most important reason for industry to not shift to bio-based production of chemicals is its higher production cost. A multi-pronged approach to constructing efficient whole-cell biocatalysts and improved production processes would be required.

**Table 1:** Advantages and disadvantages of whole-cell biocatalysis in comparison with chemical catalysis

Advantages	Disadvantages
High selectivity (which can be chiral, positional, and functional group-specific)	Catalyst stability: biocatalyst is susceptible to substrate or product inhibition; inactivation may occur at high temperatures, at extremes of pH, or in organic solvents
High catalytic efficiency	The cell membrane may act as a mass transport barrier
Multi-step reactions in single strain with cofactor regeneration	Much more likely to have undesirable metabolic by-products, which may be toxic to the cells and difficult to separate
Recycling is sometimes possible Milder operational conditions Environmentally friendly	

Whole-cell catalysis approaches can broadly be classified into biotransformation (biocatalysis) and fermentation bioprocesses. In fermentations, the products are synthesized from growth substrates via the host cells’ native metabolism and are accompanied in the fermentation broth by metabolic intermediates that make downstream processing complicated [11, 12]. In biotransformations, cell growth (the enzyme manufacturing phase) and production phase are separated. Substrates are converted to the desired products by resting cells [3, 13]. The key advantages of whole-cell biocatalysis are its abilities to use cheap and abundant raw materials and to catalyse multistep reactions. Gehring et al. reported the synthesis of rhamnolipids from the cheap raw material butane using a tailored whole-cell biocatalyst. The AlkBGT system from *Pseudomonas putida* (for butane activation) and the RhlABC system from *Pseudomonas aeruginosa* (for rhamnolipid assembly) were integrated into the cell strain to result in rhamnolipid biosynthesis that used butane as the sole carbon and energy source. This approach represents a one-pot convergent total synthesis with more than 25 steps [14]. Whole-cell biocatalysts that include active enzymes or pathways make the time-consuming and material-intensive enzyme purification process more efficient. In addition to this upstream simplification, downstream processing can also be simplified, further decreasing environmental and economic costs [13]. In an economic evaluation of a process, the cost of the product is considered. The cost of a product (US$/kg) = A + B/Yield + C/Pv, where A is the capital cost, B is the raw material cost and C is the operating cost. Here, Pv is the volumetric productivity in the unit of g/L/h. Cheap raw materials, efficient bioconversion, and the ability to reuse the biocatalyst many times reduce the cost of product and make whole-cell biocatalysis very cost-competitive with fermentation [12, 15–19].

Efficient whole-cell biocatalysts are very important for an economically feasible biocatalysis process with optimal titre, yield and productivity. The principles for designing whole-cell biocatalysts for bioconversions are quite different from those for designing the microbial cell factories that are used in fermentations [15–19]. Single or multiple enzymes, depending on the complexity of the synthetic pathway that produces the target products from the feedstock, need to be introduced into host cells to construct whole-cell biocatalysts for the production of value-added chemicals. It is important to consider whole cells, in their entirety, as catalysts and not only focus on the individual active enzymes to fully exploit the synthetic potential of microbial biocatalysts. Maximizing the flux through a synthetic pathway plays a pivotal role in obtaining the best volumetric productivity of a bioconversion, which thus lowers the production cost of the targeted chemicals. The emerging tools of synthetic biology, integrated with comprehensive omics data, facilitate metabolic engineering of microbial cells at an unprecedented level, holding promise for the development of a balanced, stable, productive, and efficient whole-cell workhorse platform [20, 21].

*Escherichia coli* is perhaps the most widely used microbial platform for cell factories. This dominance is largely because of its well-studied genetic background, a mature and powerful genetic toolset for metabolic engineering, and relatively well-developed fermentation processes with low-cost raw materials [12, 13, 16]. Whole-cell biocatalysis has even been implemented by coupling two recombinant strains [22–24]. The catalytic efficiency of such processes is low due to high mass transfer resistance, and the processes are generally too complex for use on an industrial scale [25, 26]. Assembling the synthetic pathways in a single strain reduces the mass transfer problem by avoiding the transfer of intermediates and thus greatly simplifies the operation [16, 27]. Whole-cell biocatalysis using engineered *E. coli* seems to be the most promising method and offers the potential for large-scale and low-cost production. Consequently, this review focuses on whole-cell biocatalysis using microbial cell factories based on *E. coli*.

Biocatalysis using a single strain as the sole catalytic unit can offer a broad scope of substrates that is not limited to natural pathways and thus opens the door to versatile multistep biocatalysis. Additionally, whole-cell biocatalysts can be rationally designed and readily tailored to their specific applications. In this review, we summarize the different strategies and efforts that have been used in designing and optimizing various whole-cell biocatalysts to convert cheap feedstocks to value-added chemicals.

## Advantages of whole-cell biocatalysis

### Efficiency

Whole-cell biocatalysts allow for the facile implementation of enzymatic cascades that span multiple reactions, with an integrated supply of the myriad cofactors that are needed for such complex biotransformations [12]. This internal supply greatly simplifies cofactor regeneration and makes the addition of expensive external cofactors unnecessary. Furthermore, the presence and protective nature of the cellular envelope helps stabilize the enzymes and can enable enzyme applications under harsh reaction conditions [16, 28]. Moreover, the close proximity of reactants and catalysts, as well as the inherent presence of what would otherwise be expensive external cofactors, greatly improves biocatalyst efficiency [12, 28].

### Catalyst cost

As with any catalytic process, the cost and stability of the catalyst are highly relevant to its economical application in chemical manufacturing [2]. The use of whole cells circumvents the need for cell lysis and enzyme purification that is associated with biocatalysis with isolated enzymes and inherently greatly reduces the catalyst cost. No external cofactors are needed because the expensive cofactors can be supplied and regenerated by the cell, which also reduces the cost. Whole-cell biocatalysts are generally more readily prepared, the cost of fermentation is usually not prohibitive, and furthermore, the cells can often be used repeatedly [12, 16]. Therefore, whole-cell biocatalysts have outstanding inherent cost advantages.

### Downstream processing

Typical biotransformation processes comprise two stages: growth of the living “catalyst” and conversion of the substrate(s). After cells are cultured, they are harvested and washed with water or a buffer solution and suspended in the desired buffer for biocatalysis. When the cells are washed, unconsumed growth substrates and nutrients, as well as undesired metabolites that were produced during growth, are removed from the system, allowing significantly better product recovery rates and greatly simplifying downstream processing [28]. Furthermore, the removal of necessary nutrients arrests cell growth, and resting cells can produce higher yields from their carbon source since the available carbon and energy is overwhelmingly used for product synthesis instead of biomass production [13].

## Whole-cell biocatalyst design principles

**Fig. 1:**
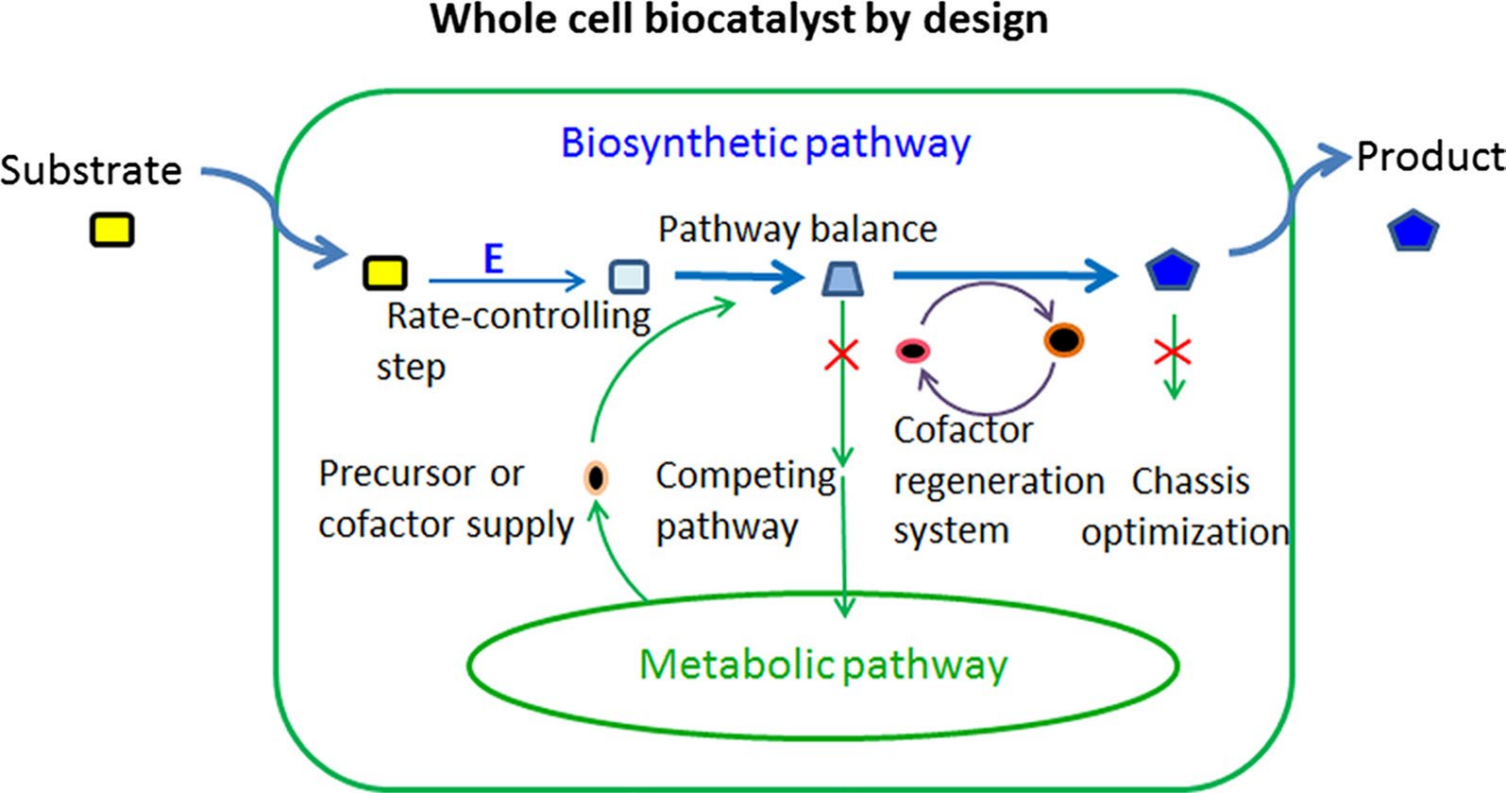
Strategies to improve whole-cell biocatalysis. Identification and relief of bottlenecks, pathway balancing to maximize flux towards the product, blocking competing pathways, improving the precursor supply, engineering cofactor or co-substrate balance and chassis optimization

The construction of effective whole-cell biocatalysts requires that single or multiple enzymes are introduced into host cells to construct synthetic pathways for the conversion of the desired feedstocks into the targeted products. Metabolic engineering and synthetic biology efforts aim to enable rational design and construction of biosynthetic pathways that maximize the pathway flux to products by providing preoptimized chassis cells that enhance the production of target compounds. The strategies that are employed in the design and optimization of whole-cell biocatalysts are discussed below (Fig. 1).

### Design and construction of the biosynthetic pathways

Many new genes and pathways have been discovered with the recent advances in genomics, proteomics, and bioinformatics. The numerous potentially valuable enzymes and pathways that are present in nature are being identified at an increasing pace [2, 12], and these enzymes and pathways may represent the most valuable resource for reconstructing synthetic pathways to produce specific molecules; their importance emphasizes the industrial and commercial need for continued funding for basic science. The identified pathways can be transferred to microbial hosts such as *E. coli* to generate heterologous biosynthesis pathways for the production of exogenous and non-natural molecules. Heterologous expression of genes from the designed pathway in *E. coli* can be controlled independent of the host’s regulatory network [12, 20], resulting in easy control of the product-forming pathway. Here, we describe the basic design principles recently used to construct single- and multistep biosynthesis pathways, including de novo biosynthetic pathways, of various chemicals.

### Single-step pathways

Although whole-cell biocatalysis is primarily an alternative for in vitro multienzyme cascades, a number of successful and commercially viable whole-cell biocatalysts that use single-step reactions to produce fine chemicals exist [13, 29]. For whole-cell biocatalysis via single-step “pathways”, recombinant plasmid-based expression of the enzyme results in high expression levels (overexpression) and thus make large amounts of the recombinant enzyme available. Whole-cell biocatalysis for one-step synthesis is especially preferred to catalysis by the isolated enzyme when the enzyme is unstable in vitro or requires expensive co-substrates or cofactors [29, 30]. In these cases, enzymes should be screened for high activity, efficient heterologous expression, and a method of blocking the catabolism of the substrate and product, if present in the host cells, as they are the most important issues to be considered.

A whole-cell catalyst that expressed a novel esterase (EstK1) in *E. coli* produced cinnamyl acetate as a result of its high transesterification activity. The conversion ratio of cinnamyl alcohol reached 94.1% at 1 h and was even higher (97.1%) at 2 h [31]. For whole-cell biocatalysis of l-carnosine production, two enzymes (DmpA from *Ochrobactrum anthropi* and BapA from *Sphingosinicella xenopeptidilytica* 3-2W4) were screened. High yields of l-carnosine (up to 71%) were obtained by optimizing relevant reaction conditions for the best-performing recombinant strain (*E. coli* DmpA_syn_). The biocatalyst had a high stability and could be used in at least five sequential batches. In another case, whole-cell biocatalysts for γ-aminobutyric acid (GABA) production were developed by overexpressing glutamate decarboxylase (GAD; EC 4.1.1.15). GADs from different species were expressed and screened, and the enzyme from *Lactococcus lactis* was chosen. The gene *gadAB* was subsequently deleted from the genome of the *E. coli* host to prevent the degradation of GABA. As a result, 614.15 g/L GABA was produced with a high molar yield (over 99%) [30]. In this case, the engineered whole-cell biocatalyst stabilizes the enzyme and reduces the degradation of the product under the reaction conditions.

Proline-4-hydroxylase, which catalyses the biotransformation of l-proline to trans-4-hydroxy-l-proline (Hyp), is a 2-oxoglutarate (2-OG)-dependent oxygenase [32]. The proline-4-hydroxylase gene from *Dactylosporangium* sp. RH1 was overexpressed in a *putA*-deficient *E. coli* mutant that cannot degrade l-proline. The co-substrate 2-OG, an intermediate of the citric acid cycle, was supplied by glucose via the central carbon metabolism of the cells. Hyp was accumulated to a final concentration of 41 g/L with a productivity of 0.41 g/L/h [32]. In this case, using the recombinant strain as whole-cell biocatalyst avoided the extrinsic addition of the expensive co-substrate 2-OG. In whole-cell biocatalysis for 2-OG production, l-glutamate oxidase (LGOX) catalyzes the transformation of l-glutamic acid to 2-OG along with the production of NH_3_ and H_2_O_2_. In order to remove H_2_O_2_, catalase was co-expressed with l-glutamate oxidase, which substantially enhanced the efficiency of 2-OG production. 77.4 g/L 2-OG with a conversion rate of 98.5% was obtained in 12 h [33].

### Multi-step biosynthesis pathways

In cases of complex multistep bioconversions, entire metabolic pathways can be transferred to the host from other organisms. The aromatic alcohol 2-phenylethanol (2-PE) is synthesized from l-phenylalanine (l-Phe) via the three-step Ehrlich pathway in yeast and via the phenylacetaldehyde synthase (PAAS) pathway in plants. The reconstitution of the Ehrlich pathway in *E. coli* introduced the enzymes that perform the necessary decarboxylation and reduction steps into the bacterial cells and resulted in 2-PE production. Approximately 96% of the final product was produced from l-phenylalanine (based on the initial 40 mM l-phenylalanine) using the recombinant *E. coli* [34]. In another case, a novel pathway that used the PAAS from *Rosa hybrid*, a pyridoxal 5′-phosphate (PLP)-dependent enzyme and endogenous alcohol dehydrogenases was introduced into *E. coli* to produce 2-PE. This biotransformation, which was based only on internal de novo PLP synthesis, produced 0.34 g/L 2-PE [35, 36].

The construction of de novo biosynthetic pathways denotes the assembly of genes from different non-related organisms to construct artificial pathways in the desired host. This approach enables retrosynthetic pathway design and opens the door to the development of unprecedented multistep biocatalysts. The seven-step metabolic pathway from glucose-6-p to N-acetyl-d-neuraminic acid (Neu5Ac) in bacteria has been identified. Although researchers have tried to metabolically engineer a corresponding *E. coli* strain, only 1.5 g/L Neu5Ac was obtained by fermentation [37]. In contrast, a de novo two-step biosynthetic pathway for Neu5Ac was designed and assembled in a single *E. coli* strain; this new pathway co-expressed GlcNAc 2-epimerase (EC 5.1.3.8, AGE) from cyanobacteria and Neu5Ac aldolase (EC 4.1.3.3, NanA) [16, 38, 39] or Neu5Ac synthase (EC 4.1.3.19, NeuB) from bacteria [23, 25, 40]. Whole-cell biocatalysts were developed for Neu5Ac production by assembling a heterologous biosynthetic pathway in *E. coli.* Ishikawa et al. constructed a recombinant *E. coli* N18-14 by overexpressing genes of GlcNAc 2-epimerase (s*lr1975*) and *neuB* resulting in a yield of 53 g/L of Neu5Ac (2.41 g/L/h) after 22 h [25]. Recently, recombinant *E. coli* co-expressing *slr1975* and *nanA* for Neu5Ac production was reported and Neu5Ac accumulated at 59 g/L after 36 h (1.64 g/L/h) [39] and 61.3 g/L in 60 h [38]. Recombinant *E. coli* consisting of AGE from *Anabaena* sp. PCC7120 and NanA from *E. coli* was used as whole-cell biocatalyst [16]. A yield of 74.2 g/L was achieved with a productivity of 6.2 g Neu5Ac/L/h. The engineered strain could be reused in at least five cycles with a productivity of >6 g/L/h [16].

Once a de novo engineered pathway has been designed and demonstrated, it can be integrated into existing platforms for secondary metabolite production in two different ways: (1) the de novo pathway can be extended by connecting it to other pathways, and (2) biocatalysts can be used to produce product derivatives by starting from chemically modified substrates. For example, Neu5Ac is the precursor of polysialic acid (PSA) and sialylated oligosaccharides. Thus, an enhanced Neu5Ac biosynthetic module can also improve production of PSA and sialylated oligosaccharides [41, 42]. Whole-cell biocatalyst that was designed for Neu5Ac has been used to produce 11 Neu5Ac derivatives using chemically modified GlcNAc analogues as substrates [16, 43]. The synthesis of optically pure secondary epoxy alcohols from racemic allylic alcohols using a whole-cell biocatalyst that is composed of recombinant *E. coli* co-expressing a styrene monooxygenase and two alcohol dehydrogenases was described by Liu et al. [44]. With the successful establishment of both the 2S and 2R systems for substrate (*rac*)-1a, this approach was extended to other substrates. As a result, excellent enantio- and diastereoselectivity were achieved for most of the 12 substrates [44]. Finally, it is conceivable that strains that improve production of a wide variety of derivatives can be obtained via protein engineering of the target enzymes.

### Improving whole-cell biocatalysts by metabolic engineering

The introduction or creation of biosynthetic pathways in microbial hosts has allowed the biocatalytic conversion of non-native chemicals. However, these pathways rarely function optimally when first introduced into the host organism, resulting in suboptimal yields of the desired product [45]. Thus, systematic optimization by metabolic engineering of both the specific pathways and the overall cellular chassis of the microbial cell factory is essential to enhance the biosynthesis of the target compound. In this section, we discuss the attempts at pathway flux maximization and chassis optimization using metabolic engineering. The strategies that were used to improve the engineered biosynthetic pathways included the identification of rate-controlling steps and relieving of bottlenecks, pathway balancing to eliminate the accumulation of toxic intermediates or by-products, and the maximization of the pathway’s flux towards the product by, for example, blocking competing pathways, enhancing the supply of precursors and co-substrates, and improving the balance of cofactors [2, 11–13, 46]. Metabolic engineering of the chassis is also necessary and may involve, among other approaches, increasing the cell’s uptake of substrates, reducing substrate and product degradation and enhancing product transport [2, 11, 46].

### Identification and relief of bottlenecks

Expression of foreign pathways often results in suboptimal performance due to unintended factors such as introduction of toxic metabolites or poor expression of pathway components [45]. The identification of rate-controlling steps is particularly important in pathway optimization. Once the bottleneck of the pathway is identified, its limiting power can be reduced by improving the expression of rate-controlling enzymes, substituting rate-controlling enzymes with a higher activity from other species, and modifying the enzymes by protein engineering [16, 45, 47]. Enhancing the expression of the rate-controlling enzyme is usually the easiest change to enact.

A whole-cell biocatalyst for Neu5Ac production was developed by assembling a heterologous biosynthetic pathway in *E. coli* that consisted of AGE and NanA. NanA was identified to be the rate-controlling enzyme of the engineered pathway. Efforts had been made to alleviate the NanA bottleneck by manipulating the amount of the recombinant enzyme. When the expression of NanA increased, a nine-fold increase in Neu5Ac production was achieved [16]. For the biosynthesis of polysialic acid (PSA), N-acetylneuraminate (Neu5Ac), 7-O (or 9-O)-acetyltransferase (NeuD), CMP-Neu5Ac synthetase (NeuA) and alpha-Neu5Ac alpha-2,8-sialyltransferase (NeuS) were required [48]. Overexpressing the key enzyme NeuD resulted in a threefold increase in PSA production relative to that in the parent strain [15].

### Pathway balance to maximize flux towards the product

Imbalances in pathway gene expression can lead to the accumulation of toxic intermediates or by-products, and the resulting metabolic burden on the host cells leads to suboptimal performance [47]. Targeted modifications on the gene level can optimize expression levels through codon usage, promoter and RBS optimization, the use of alternative genes, and the use of enzymes from other species [45, 49]. Maximizing the flux of a synthetic pathway plays a pivotal role in obtaining the best volumetric productivity of a bioconversion and thus lowering the production cost of the targeted chemicals.

A very illustrative example of these approaches is the optimization of the mevalonic acid (MVA) pathway to enhance isoprenoid production. The strategies aimed to balance the pathways and eliminate the accumulation of toxic intermediates in addition to maximizing the flux towards the product. A mevalonate-based isopentenyl pyrophosphate biosynthesis pathway (MVA) was introduced into an *E. coli* strain to produce large quantities of isoprenoids [47, 50, 51]. The MVA pathway was introduced into *E. coli* in the form of two synthetic operons, an “upper pathway” that converted acetyl-CoA to MVA and a “lower pathway” that produced dimethylallyl diphosphate (DMAPP) from MVA, that were a combination of bacterial and yeast enzymes [49, 52]. The upper pathway comprises two genes (MvaE and MvaS) from *Enterococcus faecalis*, while the lower pathway comprises the MVK, PMK, MVD, and IDI enzymes from *Saccharomyces cerevisiae* and *Methanosarcina mazei* [52]. The isoprene synthetic pathway was introduced by expressing the isoprene synthase IspS. Subsequently, the upper pathway was optimized by regulating the expression of the key enzyme (MvaE) via the incorporation of rare codons, and the lower pathway was enhanced by overexpressing the rate-controlling enzyme MVK [47]. Additionally, the upper pathway flux was regulated by origin replacement, and the lower pathway was integrated into the chromosome, effectively balancing the two pathways [47, 51, 53]. Metabolite analysis revealed that the accumulation of intermediates was eliminated by combining these multiple strategies, demonstrating that the pathway was balanced. As a result, the growth inhibition caused by the toxic intermediate mevalonate was relieved, and the lycopene yield increased [47, 51, 53].

Due to rapid advances in synthetic biology research, efficient techniques for the combinatorial assembly of large numbers of genes, operons and pathways are becoming readily available [54]. DNA assembly methods such as Gibson, Golden Gate, and randomized BioBrick assembly have been developed to enable easy construction of combinatorial libraries for the optimization of metabolic pathways. Recently, a DNA assembly method named oligo-linker mediated assembly (OLMA) was developed to simultaneously optimize multiple targets of a pathway [55]. This approach was used to fine-tune the lycopene synthetic pathway. The *crtEBI* genes from different species including *Pantoea ananatis, Pantoea agglomerans, Pantoea vagans* and *Rhodobacter sphaeroides* in combination with the host’s native IDI were assembled in *E. coli* to construct the basic lycopene synthesis pathway. The pathway in the library was varied by recombining four RBS targets and the *crtEBI* genes from different species and by varying the gene order. Strikingly, all of this variation was implemented in a single assembly step using the OLMA method. The library was subsequently directly analysed for lycopene production, and the enzymes of the lycopene synthesis pathway and their expression levels were optimized to result in a striking increase in yield from 0.14 to 15.17 mg/g DCW [55].

### Blocking competing pathways

Blocking the competing pathways that drain substrates and intermediates prevents their diversion from the desired biosynthesis pathway and usually increases the flux and final titre of the product, as expected. The removal of the *nanA* and *nanT* genes that encode the Neu5Ac aldolase and Neu5Ac transporter, respectively, abolished sialic acid catabolism, while knocking out the *nanK* (encoding the Neu5Ac transporter) and *nanA* genes prevented ManNAc and Neu5Ac from being diverted from the biosynthetic pathway [37, 42]. When the *nanTEK* genes of engineered *E. coli* were knocked out, Neu5Ac production was enhanced threefold, resulting in 173.8 mM Neu5Ac [16]. The combination of blocking Neu5Ac uptake and preventing the diversion of ManNAc from the desired biosynthetic pathway pushed the two reversible reactions towards the synthesis of Neu5Ac, which synergistically resulted in the production of a large amount of Neu5Ac.

### Improving the precursor supply

In engineered strains, bioconversion efficiency is determined not only by metabolic flux but also by the efficient turnover of precursors [55]. When a heterologous pathway is introduced into a production host, the pathway will unavoidably compete with the native metabolism for common precursors. Consequently, elevating the levels of important precursors by redirecting the corresponding fluxes can be an efficient strategy to enhance production of target compounds.

For 2-*C*-methyl-d-erythritol 4-phosphate (MEP)-dependent carotenoid biosynthesis, an imbalanced supply of glyceraldehyde 3-phosphate (G3P) and pyruvate precursors is one of the major metabolic bottlenecks, especially considering the limit of the G3P precursor’s availability. Systematic modification of targets within central metabolic pathways was conducted to promote the redistribution of metabolic fluxes towards MEP-dependent carotenoid biosynthesis. The flux to the EMP pathway was rewired towards the ED/PP pathways by knocking out phosphoglucose isomerase (PGI). Tweaking the flux at the branch point between ED and PP by overexpressing *eda* and fine-tuning *gnd* expression in a PGI-deficient strain (Δ*pgi*) improved the G3P/Pyr supply and rebalanced precursor availability, which relieved the bottleneck. Further improvements in DXS expression led to efficient use of G3P and pyruvate in the MEP pathway and significantly increased productivity [55]. Thus, the established flux distribution resulted in an efficient supply and optimal ratio of precursors, resulting in a stable balance between carotenoid biosynthesis and cell growth that yielded optimal overall productivity.

### Engineering cofactor or co-substrate balance

A whole-cell biocatalyst is usually preferred for cofactor-dependent reactions since the inherent presence of cofactors that are generated by the host cell and the ease of their recycling greatly improve the economics of the process [2]. In cells, the cofactors, such as nicotinamide, 2-oxoglutarate, acetyl-CoA and ATP, are mainly used in glycolysis and the citric acid cycle and are present at low concentrations. Cofactor supply and regeneration may be limited if the target biosynthetic reaction is rapid. Therefore, metabolic engineering to increase cofactor supply and regeneration is necessary. Cofactor recycling is key not only to lowering the cost of a process but also to driving the reaction of interest to completion.

**Fig. 2:**
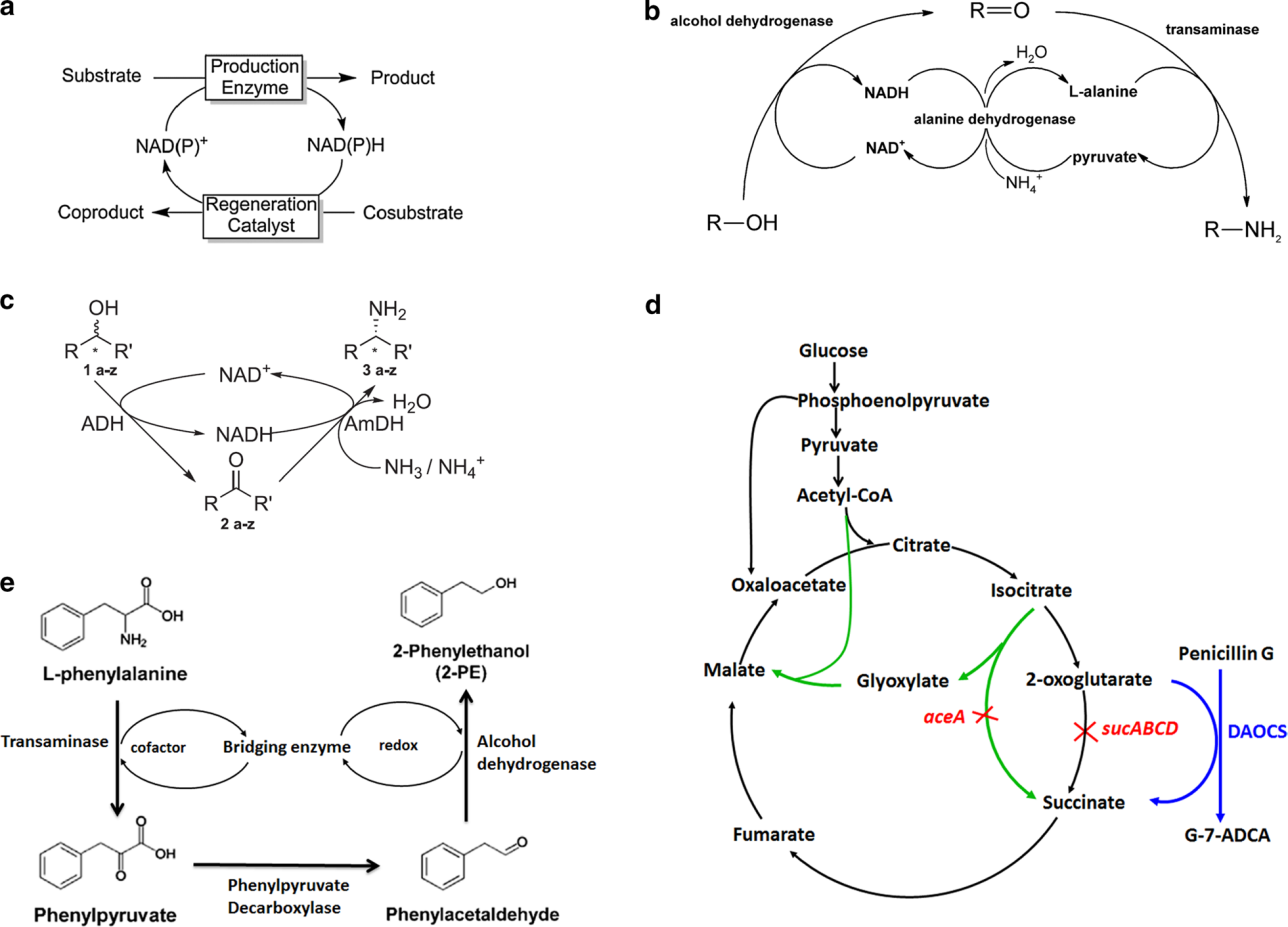
Engineering cofactor or co-substrate balance. **a** NAD(P)H regeneration systems formed via coupling with a regeneration reaction [56]; **b** redox self-sufficient amination via coupling with an alcohol dehydrogenase, l-alanine-dependent transaminase and l-alanine dehydrogenase [62, 63]; **c** redox self-sufficiency via a two-enzyme cascade for the hydrogen-borrowing amination of alcohols [54]; **d** reconstitution of TCA cycle using a DAOCS-catalysed reaction for 2-OG supply and regeneration [66]; **e** cofactor self-sufficient system established via a bridging mechanism (enzymes) to enable the simultaneous regeneration of cofactors and redox equivalent

Increased cofactor regeneration is traditionally accomplished using an in situ regeneration reaction (Fig. 2a). For oxidoreductase-catalysed reactions that depend on nicotinamide cofactors, cofactor recycling in whole-cell biocatalysts is achieved by cascading the reaction with a dehydrogenase and thus coupling the recycling of the nicotinamide cofactor to the conversion of a sacrificial co-substrate by formate dehydrogenase, glucose dehydrogenase, alcohol dehydrogenase, phosphite dehydrogenase or hydrogenase [56–59]. The dehydrogenases that are most commonly exploited for the recycling of NAD(P)H are formate dehydrogenase and glucose dehydrogenase, which obtain reduction equivalents by enzymatically oxidizing the sacrificial substrates formate and glucose, respectively [60, 61].

Recently, a self-sufficient redox system that utilizes the direct coupling of oxidizing and reducing enzymatic reactions has been developed [59, 62–64]. Thus, neither additional substrate nor another regenerating enzyme is required for this type of recycling reaction. This elegant method is applicable in special cases only, but increasing numbers of examples have been reported in the last few years [59, 62–64]. Cascading alcohol oxidation and l-alanine-dependent transamination with an l-alanine dehydrogenase enabled the self-sufficient redox conversion of alcohols to the corresponding amines (Fig. 2b) [62, 63]. Efficient self-sufficient redox amination of aliphatic and aromatic (di)alcohols was achieved in vivo so that the addition of the (very expensive) transaminase cofactor pyridoxal phosphate and the alcohol dehydrogenase cofactor NAD^+^ was not necessary to obtain complete conversion [62, 63]. Recently, Mutti et al. designed an elegant self-sufficient in vitro redox system that converted alcohols to amines using alcohol dehydrogenase (ADH) and amine dehydrogenase (AmDH) (Fig. 2c), which operated in tandem while hydrogen was shuttled by a nicotinamide coenzyme. This self-sufficient redox cascade demonstrates high atom efficiency by sourcing nitrogen from ammonium and generating water as the sole by-product, which results in an exceedingly clean system [64].

The 2-OG-dependent oxygenases have emerged as the largest known family of nonheme-oxidase enzymes and are involved in the biosynthesis of a truly vast variety of metabolites, including materials of medicinal or agrochemical importance (e.g., gibberellins and antibiotics such as cephalosporins and clavulanic acid) [65]. These reactions require 2-OG, which undergoes oxidative decarboxylation to form succinate, as a co-substrate. However, 2-OG is normally metabolized via the TCA cycle, and thus little flux normally goes into the synthesis pathway of the desired product. To address this problem, An elegant strategy had been developed by by constructing a modified TCA cycle that changed the role of 2-OG from co-substrate to cofactor and then regenerated it (Fig. 2d) [66]. In this work, *E. coli* cells expressing deacetoxycephalosporin-C synthase (DAOCS) were developed as a whole-cell biocatalyst to convert penicillin G to G-7-ADCA [66]. The TCA cycle was engineered in vivo by blocking the normal TCA reaction leading from 2-OG to succinate, effectively coupling it with the DAOCS-catalysed reaction to form a modified TCA cycle. Thus, the metabolic flux from the central metabolism was forced to go through the DAOCS-catalysed reaction to produce G-7-ADCA. This strategy was combined with other efforts including reducing the accumulation of acetate and blocking the degradation of penicillin G and G-7-ADCA, which led to an 11-fold increase of the efficiency of the whole-cell biocatalyst. This example thus demonstrates the feasibility of redirecting the TCA cycle to drive a desired enzymatic reaction—a strategy which will most certainly be applied to other products that require 2-OG in the near future.

For biosynthetic pathways that encompass both co-substrate- and redox-dependent reactions, e.g., 2-phenylethanol (2-PE) biosynthesis, cofactors such as 2-OG and NAD(P)H are required for the transamination and dehydrogenation reactions, respectively. The simultaneous regeneration of cofactors and redox equivalents remains a challenge. However, it is possible to construct a “bridge” between an amino acid and a structurally equivalent fusel alcohol by using glutamate dehydrogenase. Thus, to develop a self-sufficient cofactor system to enhance production of 2-PE in *E. coli*, the researchers coupled bridging enzymes with transaminase and alcohol dehydrogenase so that the cofactor and redox equivalents were regenerated simultaneously and no external cofactor or redox source was required [67]. A self-sufficient cofactor system based on a bridging mechanism was thus developed, improving the biocatalyst efficiency by 3.8-fold (unpublished data). This self-sufficient cofactor strategy offers a new method to resolve the cofactor/redox imbalance.

### Chassis optimization

Metabolic engineering of the chassis cell to further improve the performance of whole-cell biocatalysts may involve increasing the cells’ uptake of substrate(s), reducing substrate and product degradation, and blocking the effects of proteases to stabilize overexpressed intracellular enzymes [55, 66].

GlcNAc is transported by GlcNAc-specific PTS into cells as GlcNAc-6-P and then enters the NAG pathway to be utilized as a carbon and nitrogen source. Elimination of GlcNAc-specific PTS reduced GlcNAc-related side reactions and increased Neu5Ac production by 1.28-fold [39]. The outer membrane protein AlkL of *P. putida* GPo1 was reported to improve hydrophobic substrate uptake into *E. coli* [68]. *E. coli* cells overexpressing the monooxygenase system AlkBGT and the uptake facilitator AlkL were used as a whole-cell biocatalyst to oxyfunctionalize renewable fatty acid methyl esters [69]. However, extensive dodecanoic acid methyl ester uptake that was mediated by high AlkL levels led to whole-cell biocatalyst toxification. By fine-tuning the AlkL expression and reducing alkBGT expression, the product titre was increased from 4.3 to 229 g/L in a two-liquid phase bioprocess [69].

Reducing the degradation of substrate and product is important for increasing substrate utilization and thus increasing the overall conversion rate. For example, penicillin G and G-7-ADCA are susceptible to decomposition; knocking out the gene that encoded β-lactamase (*ampC*) resulted in a 3.9-fold increase in the G-7-ADCA production over that of the parent strain [66].

Low amounts of the relevant enzymes are obtained, which leads to suboptimal performance, if the enzymes of the synthesis pathway are susceptible to protease attack [70]. In such cases, the protease(s) that is responsible for biocatalyst degradation must be identified, possibly by screening corresponding deletion mutants and hopefully finding a suitable chassis.

## Process engineering

To develop an economically feasible whole-cell biocatalysis process, in addition to the rational design of whole-cell biocatalysts, it is of great importance to also optimize the entire production process to achieve economic viability. Whole-cell biocatalysis processes must, by definition, involve a growth process and a substrate conversion process. Cells are cultured, removed from the growth medium and then resuspended in different biotransformation media to convert substrates into desired products [3, 13]. Several considerations influence the optimal growth of the biocatalyst, as with any fermentation, but the major issues regarding their use in biotransformations are the expression level(s) of the enzyme(s) of interest and the biomass yield. For most whole-cell biocatalysts that contain multistep pathways, coordinated expression, but not necessarily overexpression of the many enzymes involved in the pathways, is of great importance [16, 41, 49, 51]. A good balance is a prerequisite for the efficiency of the biocatalyst. In single-step biotransformations, on the other hand, optimal overexpression of a single enzyme is usually sought [29, 30]. In both cases, biomass is an important factor to consider in the cell growth process since a higher biomass, especially if obtained from a cheap fermentation medium, means a lower cost of the biocatalyst.

To implement a whole-cell biotransformation, the substrate of interest must be transported across the cell membrane to reach the active enzyme or enzyme system. The same issues exist for the product. Even though the substrate can usually enter the cell by passive diffusion [28], mass transport must be considered. The mass transfer resistance is mainly caused by the cell membrane, which acts as a mass transport barrier for both the substrate and the product. Several studies have shown that it is possible to improve substrate transfer across cell walls and membranes by increasing their permeabilization level by chemical (detergents and solvents) or physical (e.g., temperature shock) means. For example, surfactants and organic solvents (Triton X-100, Tween 80, Xylene and CTAB) were added to the reaction mixture to enhance the transport of GlcNAc into cells, which resulted in improved production [16, 23, 39]. The permeabilized cells effectively had “holes” in their cellular membrane while leaving enough of the cell membrane and cell wall intact to contain the enzymes and other macromolecules [13]. This technique is especially useful when transport issues are found to be limiting.

## Conclusion and perspective

Whole-cell biocatalysts can convert cheap feedstocks into complex, value-added fine chemicals with a range of applications in pharmaceutical and chemical industries. What is more, efficient whole-cell biocatalysts can increasingly be rationally designed. The present work reviews strategies for the metabolic engineering of whole-cell biocatalysts based on the well-established *E. coli* platform. As described above, advances in metabolic engineering and synthetic biology have markedly improved the productivities and yields of products that are synthesized using whole-cell biocatalysts.

Despite the advantages outlined in this article, there are certain limitations that should be considered. The mass transport barrier that the cell membrane represents is one limitation. Cells often have a specific system to transport compounds in or out of the cell. Enhancing the transport system may help the transportation of substrates and products. Another method that can be used to improve substrate transfer across cell walls and membranes is to increase their permeabilization level by chemical means. However, these methods may damage the cell integrity and cause leakage of cellular components, complicating downstream process engineering; therefore, the best conditions for permeabilizing cells should be determined. Second, metabolic engineering strategies that are used in rational strain design involve the overexpression, deletion or down-regulation of genes in their native metabolic pathways [46, 71, 72]. However, a precise control of native gene expression levels is important for cell growth. Total deletion or overexpression of metabolic branches can sometimes result in poor growth and thus in poor biocatalyst expression, especially when the targets are in the central metabolic pathways. Several recent studies aimed at overcoming these limitations have focused on the experimental and theoretical advantages that are associated with the dynamic control of enzyme levels [72–77]. For example, a molecular switch for the dynamic control of gene expression is expected to activate target gene expression in the cell growth phase and deactivate it in the bioconversion stage. The whole-cell biocatalyst will be in its optimal state for cell growth and for efficient biotransformation when such dynamic control is used.

Whole cell biocatalysis has been successful in bioconverting non-native substrates into target products. However, challenges still remain when whole-cell biocatalysis uses glucose as a substrate for fine chemical production. Although cells are in a resting state in the conversion stage, the enzymes of central metabolism are still active; thus, the introduced biosynthesis pathways compete with the native enzymes of the central pathways for substrates and energy. Metabolically engineering targets in the central metabolic pathways would affect cell physiology and lead to fluctuations in cell growth. Redirecting fluxes of central metabolism to cell growth or to biosynthesis pathways through dynamic control of native enzyme expression are expected to solve the problem [72–77]. Dynamic gene expression profiles enable better management of the balance between growth and chemical production and can thus help avoid accumulation of undesired intermediates.

The increasingly sophisticated synthetic biology and metabolic engineering toolbox is already having an impact on the number and frequency of reported successful whole-cell biocatalytic processes. This field will thus be highly dynamic for the foreseeable future.

## Abbreviations

*E. coli*: *Escherichia coli*
TCA: tricarboxylic acid cycle
ED: Entner–Doudoroff pathway
PP: pentose phosphate pathway
NAD(P)H: nicotinamide adenosine dinucleotide (phosphate)
7-ADCA: 7-aminodeacetoxycephalosporanic acid
